# Acute-on-Chronic Liver Failure: An Eroded Cliff Hit by a Storm—A Narrative Review

**DOI:** 10.3390/ijms27146414

**Published:** 2026-07-19

**Authors:** Kinga Knop-Chodyła, Beata Kasztelan-Szczerbinska, Halina Cichoż-Lach

**Affiliations:** 1 Doctoral School of the Medical University of Lublin, Medical University of Lublin, Witolda Chodźki 7, 20-093 Lublin, Poland; kinga.knop03@gmail.com; 2 Department of Gastroenterology and Hepatology with Endoscopy Unit, Medical University of Lublin, Jaczewskiego 8, 20-090 Lublin, Poland; halina.lach@umlub.edu.pl

**Keywords:** acute-on-chronic liver failure, cirrhosis-associated immune dysfunction, immunometabolism, gut–liver axis, liver–spleen axis, plasma exchange, liver transplantation

## Abstract

Acute-on-chronic liver failure (ACLF) is a rapidly progressing and highly lethal clinical syndrome characterized by multiorgan failure, driven primarily by a severe systemic inflammatory response. The pathophysiological cascade, triggered by a “cytokine storm,” subsequently evolves into profound immune paralysis. This phenomenon is driven by the dysfunction of monocytes, neutrophils, and other immune cells, compounded by their impaired cellular energetics resulting from a metabolic shift toward less efficient energy-yielding mechanisms, mainly aerobic glycolysis, with the pentose phosphate pathway contributing NADPH and biosynthetic precursors rather than ATP. This process is further exacerbated by disruptions within the gut–liver axis, wherein severe dysbiosis and impaired intestinal barrier integrity promote pathogen translocation. Beyond the gut, the liver–spleen axis constitutes a second amplification loop: the congested and immunologically remodeled spleen is proposed to sustain portal hypertension, to contribute to the circulating cytokine pool and to relay profibrogenic signals back to the liver. Coupled with generalized endothelial dysfunction, this is thought to contribute to the failure of peripheral organs. This cascade is presented as a synthesizing model of partially overlapping mechanistic hypotheses and heterogeneous evidence—much of it derived from studies in cirrhosis or animal models and still requiring deeper, ACLF-specific investigation rather than a fully established, strictly linear sequence. To date, no specific targeted therapies are available, and liver transplantation remains the sole intervention capable of substantially improving patient prognosis. Experimental immunomodulatory approaches including granulocyte colony-stimulating factor (G-CSF), intravenous albumin supplementation, therapeutic plasma exchange, mesenchymal stem cell therapy, and anti-cytokine agents represent promising therapeutic avenues. Nevertheless, appropriately tailoring these interventions to the evolving pathophysiological phases of the disease remains a significant clinical challenge, underscoring the critical need for developing precision therapies targeted at specific molecular pathways.

## 1. Introduction

Acute-on-chronic liver failure (ACLF) is a relatively recently defined, highly lethal clinical syndrome that develops in patients with chronic liver disease (with or without cirrhosis). It is distinguished by the rapid development of multiorgan failure (including the liver and at least one of the following five organs or systems: urinary system, central nervous system, respiratory system, cardiovascular system, and coagulation system). The short-term mortality associated with this syndrome remains dramatically high, ranging from approximately 20–30% at ACLF-1 to over 70% at ACLF-3 at 28 days [1,2]. As demonstrated by the results of the European PREDICT study, ACLF is not merely the end stage of decompensation but a distinct pathophysiological entity with a different clinical course and prognosis [3]. Although various geographically conditioned definitions of this syndrome exist in the literature, its pathophysiological foundation is of a common origin [4,5]. The diagnostic criteria adopted by different consortia are summarized in Table 1.

More precisely, under the European Association for the Study of the Liver—Chronic Liver Failure Consortium (EASL-CLIF) criteria an isolated kidney failure is itself sufficient to define ACLF, whereas a single non-renal organ failure qualifies only when accompanied by concurrent renal and/or cerebral dysfunction; the 2025 EF-CLIF international consensus has since standardized the definitions of liver, kidney, brain, coagulation, digestive, circulatory and respiratory failure, together with the major precipitants, to harmonize how ACLF is identified and treated worldwide [7]. Critically, the 2025 Asian Pacific Association for the Study of the Liver (APASL) Kyoto Consensus represents one of the first efforts to harmonize these regional definitions globally convening APASL, European Association for the Study of the Liver (EASL) and American Association for the Study of Liver Diseases (AASLD) stakeholders and reframes these previously parallel, unreconciled consortium definitions into a single typology rather than presenting them as separate silos: Type A ACLF corresponds to the APASL/Japanese construct (a first acute hepatic insult without prior decompensation or extrahepatic injury, potentially reversible, with an approximately 30–40% 28-day mortality), whereas Type B ACLF encompasses the AASLD, EASL-CLIF, NACSELD definitions arising on already decompensated cirrhosis; the HBV-related COSSH definition, which typically develops on non-decompensated chronic hepatitis B, aligns predominantly with Type A, consistent with Table 1. Drawing on the approximately 10,500-patient AARC database, the Kyoto Consensus also formally introduces the ‘Golden Therapeutic Window’, the ‘transplant window’ and therapeutic plasma exchange as a recognized treatment modality; Table 1 is therefore presented within this unified framework [2,7].

The majority of ACLF episodes are precipitated by defined triggering factors, which exhibit distinct geographical variability. While severe alcohol-associated hepatitis and bacterial infections predominate in Western populations, the most common trigger in Asian countries is the reactivation of hepatitis B virus (HBV) infection [3,6]. It is worth emphasizing, however, that in approximately 30–40% of patients, a definitive precipitating factor cannot be clearly identified [1].

Central to the pathophysiology of ACLF is the development of extremely severe systemic inflammation (SI), which is initiated by two groups of signaling molecules. The first group comprises pathogen-associated molecular patterns (PAMPs)—products of bacterial origin that enter the circulation as a result of translocation across a compromised intestinal barrier. The second group consists of damage-associated molecular patterns (DAMPs), which are released following tissue injury, including massive hepatocyte necrosis [8]. These molecules stimulate pattern recognition receptors (PRRs) (e.g., Toll-like receptors [TLRs] and NOD-like receptors [NLRs]) located on the surface of innate immune cells, such as Kupffer cells, neutrophils, and monocytes [9]. This leads to their uncontrolled hyperactivation and triggers a phenomenon termed a “cytokine storm,” characterized by the massive secretion of proinflammatory mediators, including interleukin 6 (IL-6), interleukin 8 (IL-8), interleukin 1 beta (IL-1β), and tumor necrosis factor alpha (TNF-α) [9].

The progressive impairment of defense mechanisms begins as early as the stage of compensated liver cirrhosis. As the ACLF syndrome progresses, driven by accumulating metabolic disturbances and the exhaustion of immunological pathways, this impairment reaches an extreme intensity, resulting in a profound impairment of immune cell function [10]. This immune paralysis is evidenced by significantly elevated concentrations of anti-inflammatory cytokines, primarily interleukin 10 (IL-10), which, by inhibiting the nuclear factor kappa B (NF-κB) pathway, blocks further transcription and secretion of proinflammatory cytokines [9]. Furthermore, immune cells are characterized by the overexpression of inhibitory receptors—MER receptor tyrosine kinase (MERTK) on the surface of monocytes, as well as programmed cell death protein 1 (PD-1) and T-cell immunoglobulin and mucin-domain containing-3 (TIM-3) on lymphocytes, along with a substantial decrease in the production of interferon gamma (IFN-γ), which additionally exacerbates immune system dysfunction [11].

**Figure 1:**
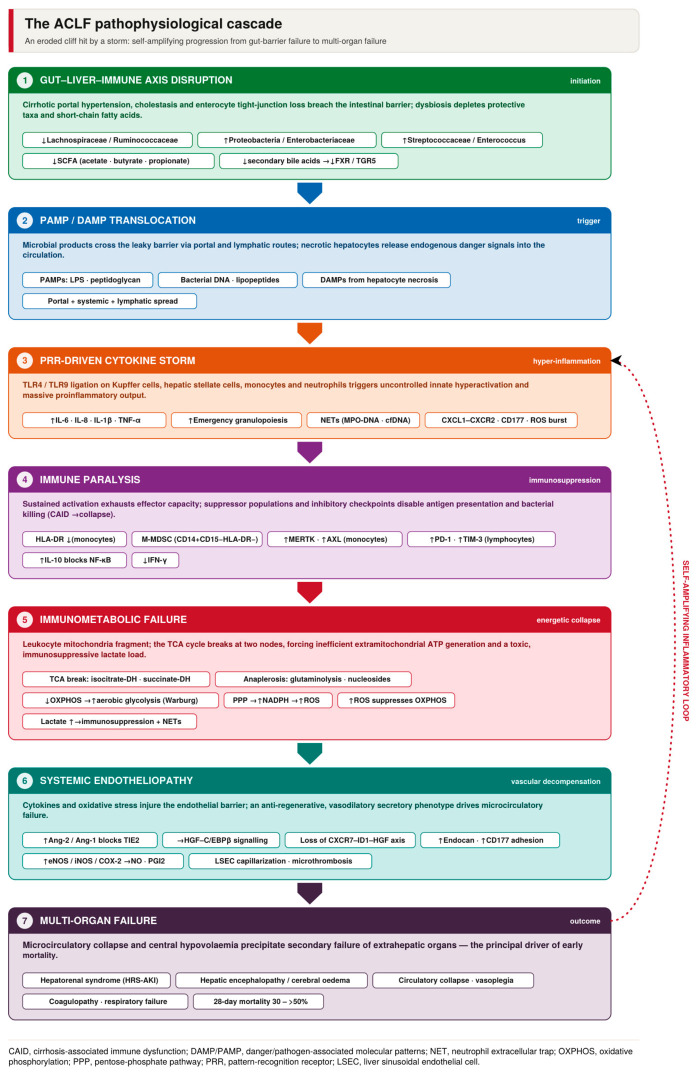
The ACLF pathophysiological cascade. Schematic of the sequential, self-amplifying mechanisms that drive acute-on-chronic liver failure (ACLF), read top to bottom as seven linked stages. (1) Gut–liver–immune axis disruption: cirrhotic portal hypertension, cholestasis and enterocyte tight-junction loss breach the intestinal barrier, with dysbiosis (loss of Lachnospiraceae/Ruminococcaceae; expansion of *Proteobacteria*, Enterobacteriaceae and *Streptococcaceae*) and depletion of protective short-chain fatty acids. (2) PAMP/DAMP translocation: microbial products cross the leaky barrier via portal and lymphatic routes while necrotic hepatocytes release endogenous danger signals into the circulation. (3) PRR-driven cytokine storm: engagement of pattern-recognition receptors on Kupffer cells, hepatic stellate cells, monocytes and neutrophils triggers uncontrolled innate hyperactivation and a mediator surge (IL-6, IL-8, IL-1β, TNF-α; emergency granulopoiesis; NETs; DAMPs). (4) Immune paralysis: sustained activation exhausts effector capacity, expands suppressor populations and engages inhibitory checkpoints, disabling antigen presentation and bactericidal killing (reduced monocyte HLA-DR and MERTK; T-cell TIM-3; reduced IFN-γ). (5) Immunometabolic failure: leukocyte mitochondrial fragmentation breaks the tricarboxylic-acid (TCA) cycle at key nodes, forcing inefficient extramitochondrial ATP generation and a toxic, immunosuppressive lactate load. (6) Systemic endotheliopathy: cytokines and oxidative stress injure the endothelial barrier, and an anti-angiogenic, vasodilatory secondary phenotype drives microcirculatory failure. (7) Multiorgan failure: microcirculatory collapse and central hypovolaemia precipitate secondary failure of extrahepatic organs—the principal driver of early mortality (hepatorenal syndrome [HRS-AKI], hepatic encephalopathy/cerebral oedema, circulatory collapse/vasoplegia, coagulopathy/respiratory failure; 28-day mortality 30 → 50%). The vertical arrow denotes the self-amplifying inflammatory loop that links the stages. Abbreviations: CAID, cirrhosis-associated immune dysfunction; DAMP/PAMP, danger/pathogen-associated molecular patterns; HLA-DR, human leukocyte antigen–DR; IFN, interferon; IL, interleukin; NET, neutrophil extracellular trap; OXPHOS, oxidative phosphorylation; PPP, pentose–phosphate pathway; PRR, pattern-recognition receptor; LSEC, liver sinusoidal endothelial cell; TCA, tricarboxylic-acid cycle; TNF, tumour necrosis factor.

The consequences of such severe immunological impairment extend far beyond the hepatic environment. Circulating cytokines damage the endothelial barrier, induce profound oxidative stress, and generate severe microcirculatory disturbances alongside adverse changes in the metabolism of distant tissues. This pathway leads directly to secondary dysfunction and, ultimately, the failure of extrahepatic organs, which constitutes the primary cause of early mortality in these patients [12,13]. The ACLF pathophysiological cascade is presented in Figure 1.

The novelty of this review lies in integrating the newly harmonized 2025 APASL Kyoto and EF-CLIF definitions with an immunometabolic perspective, the gut–liver–immune axis and endothelial injury, and in synthesizing these strands into a phase-targeted therapeutic framework that aligns each intervention with the dominant immune state. Such an integration has not been offered by prior ACLF reviews that treat these domains separately.

## 2. Methods

**Figure 2:**
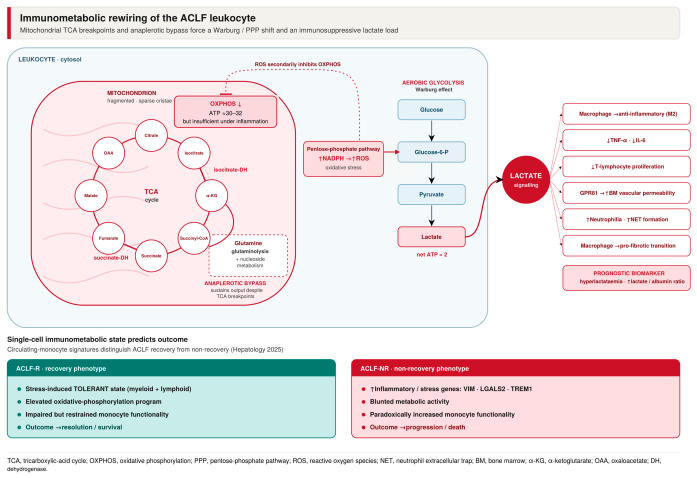
The immunometabolic rewiring of the ACLF leukocyte. Mitochondrial TCA-cycle breakpoints and anaplerotic bypass force a Warburg-like shift toward aerobic glycolysis and an immunosuppressive lactate load in the ACLF leukocyte. Left (leukocyte cytosol): the mitochondrion is fragmented with sparse cristae; oxidative phosphorylation (OXPHOS) is suppressed (ATP ≈ 30–32 but insufficient under inflammation) and reactive oxygen species (ROS) further inhibit OXPHOS. The tricarboxylic-acid (TCA) cycle is interrupted, with anaplerotic bypass through glutaminolysis (glutamine → α-ketoglutarate) and nucleoside metabolism sustaining partial output. Centre: carbon flux is diverted through aerobic glycolysis (glucose → glucose-6-phosphate → pyruvate → lactate; net ATP = 2) with pentose–phosphate pathway (PPP) shunting (increased NADPH and ROS). Right: the resulting lactate signalling promotes an anti-inflammatory (M2) macrophage programme, suppresses TNF-α/IL-6 and T-lymphocyte proliferation, increases GPR81-mediated bone-marrow vascular permeability, and drives neutrophil NET formation and a pro-fibrotic macrophage transition; hyperlactataemia and a low lactate:albumin ratio serve as prognostic biomarkers. Bottom: single-cell immunometabolic state predicts outcome—Circulating-monocyte signatures distinguish an ACLF-recovery phenotype (stress-induced tolerant state [myeloid + lymphoid], elevated oxidative-phosphorylation programme, impaired but restrained monocyte function; resolution/survival) from a non-recovery phenotype (inflammatory/stress genes VIM, LGALS2, TREM1; blunted metabolic activity; paradoxically increased monocyte function; progression/death). Abbreviations: TCA, tricarboxylic-acid cycle; OXPHOS, oxidative phosphorylation; PPP, pentose–phosphate pathway; ROS, reactive oxygen species; NET, neutrophil extracellular trap; BM, bone marrow; α-KG, α-ketoglutarate; OAA, oxaloacetate; DH, dehydrogenase; GPR81, G-protein-coupled receptor 81; IL, interleukin; TNF, tumour necrosis factor.

**Figure 3:**
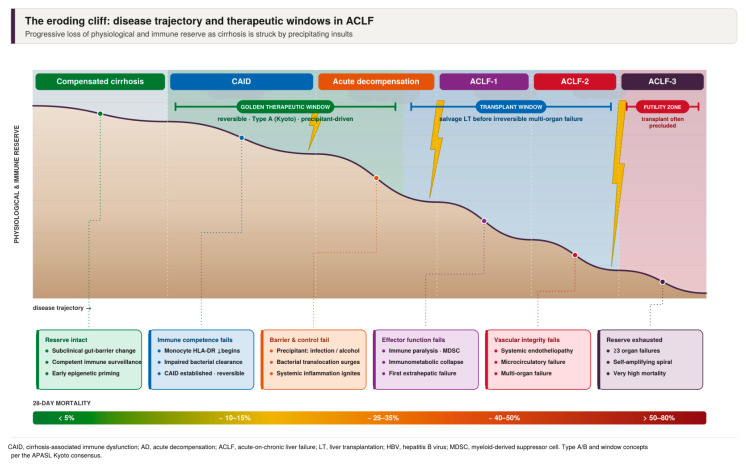
The disease trajectory and therapeutic windows in ACLF. “Eroding-cliff” representation of the progressive, stepwise loss of physiological and immune reserve (vertical axis) as a cirrhotic patient is struck by precipitating insults along the disease trajectory (horizontal axis, left to right): compensated cirrhosis → cirrhosis-associated immune dysfunction (CAID) → acute decompensation (AD) → ACLF grades 1–3. Two intervention windows are highlighted: the golden therapeutic window (reversible, Type A [Kyoto], precipitant-driven disease) and the transplant window (salvage liver transplantation [LT] before irreversible multiorgan failure), beyond which lies a futility zone where transplantation is often precluded. The boxes beneath each stage summarise the dominant biology: reserve intact (subclinical gut–barrier change, competent immune surveillance, early epigenetic priming); immune competence fails (declining monocyte HLA-DR, impaired bacterial clearance, CAID established and still reversible); barrier and control fail (precipitant infection/alcohol, bacterial-translocation surge, ignition of systemic inflammation); effector function fails (immune paralysis, MDSC, immunometabolic collapse, first extrahepatic failure); vascular integrity fails (systemic endotheliopathy, microcirculatory failure, multiorgan failure); and reserve exhausted (≥3 organ failures, self-amplifying spiral, very high mortality). The graded colour bar indicates rising 28-day mortality (<5% to >50–80%) across the trajectory. Abbreviations: CAID, cirrhosis-associated immune dysfunction; AD, acute decompensation; ACLF, acute-on-chronic liver failure; HLA-DR, human leukocyte antigen–DR; LT, liver transplantation; HBV, hepatitis B virus; MDSC, myeloid-derived suppressor cell. Type A/B and window concepts follow the APASL Kyoto consensus.

**Figure 4:**
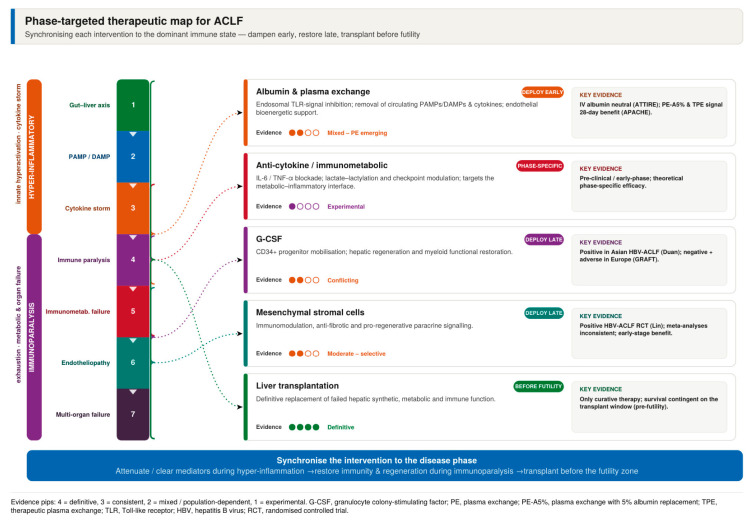
The phase-targeted therapeutic map for ACLF. Map aligning candidate ACLF therapies to the dominant immune state of the syndrome—Dampening mediators early, restoring immunity and regeneration late, and transplanting before futility. The left spine tracks the seven pathophysiological phases of Figure 1, grouped into a hyperinflammatory band (gut–liver axis, PAMP/DAMP, cytokine storm) and an immunoparalysis band (immune paralysis, immunometabolic failure, endotheliopathy, multiorgan failure). Each therapy is connected to the phase(s) it targets and is annotated with its mechanism, an evidence rating (filled pips: 4 = definitive, 2 = mixed/population-dependent, 1 = experimental) and key trial evidence: albumin and plasma exchange (endosomal TLR-signal inhibition; removal of circulating PAMPs/DAMPs and cytokines; endothelial support—Deploy early; mixed, PE emerging; intravenous albumin neutral [ATTIRE], PE-A5%/TPE 28-day signal [APACHE]); anti-cytokine/immunometabolic agents (IL-6 and TNF-α blockade; lactate–lactylation and checkpoint modulation targeting the intersection of metabolism and inflammation at each disease phase; experimental; preclinical/early-phase); G-CSF (CD34+ progenitor mobilisation; hepatic regeneration and myeloid functional restoration—Deploy late; conflicting; positive in Asian HBV-ACLF [Duan] but negative/adverse in Europe [GRAFT]); mesenchymal stromal cells (immunomodulatory, anti-fibrotic and pro-regenerative paracrine signalling—Deploy late; moderate/selective; positive HBV-ACLF RCT [Lin], meta-analyses inconsistent); and liver transplantation (definitive replacement of failed hepatic synthetic, metabolic and immune function—Deploy before futility; definitive; the only curative therapy, with survival contingent on the transplant window). The banner restates the unifying principle: synchronise each intervention to the disease phase. Abbreviations: G-CSF, granulocyte colony-stimulating factor; PE, plasma exchange; PE-A5%, plasma exchange with 5% albumin replacement; TPE, therapeutic plasma exchange; TLR, Toll-like receptor; HBV, hepatitis B virus; RCT, randomised controlled trial; IL, interleukin; TNF, tumour necrosis factor; MSC, mesenchymal stromal cell.

The aim of this narrative review is to comprehensively present the principal cellular and molecular pathophysiological mechanisms underlying ACLF, with a particular emphasis on immune paralysis, cellular metabolic dysfunction, and the gut–liver–immune axis, as well as to discuss possible future therapeutic directions. A comprehensive review of the current literature was conducted by searching for publications from 2016 to 2026 (final literature search: 30 June 2026). Titles and abstracts were screened independently by two authors, and studies were prioritized in the following order: international guidelines and consensus statements, randomized controlled trials, and the largest cohort studies; seminal pre-2016 works (e.g., the CANONIC study) were additionally included by hand-searching the reference lists of retrieved articles. As a narrative review, this synthesis is qualitative and does not report a formal PRISMA record count. The search strategy employed the following keywords: “acute-on-chronic liver failure”, “ACLF”, “advanced liver disease”, “humoral mediators”, “immune cells”, “immunosuppression”, “metabolism”, “systemic inflammation”, “immunometabolism”, and “cytokines”. The electronic databases searched included Embase, PubMed and Scopus. To be included in the analysis, studies were required to focus on one or more of the following domains: immune system dysfunction, metabolic disorders, immune cell dysfunction, the role of the gut–liver axis and the microbiome, endothelial dysfunction, and immunomodulatory therapeutic strategies in ACLF. Eligible article types comprised original research, meta-analyses, systematic reviews, and official guidelines from international hepatology societies published in English. Both human and animal studies were considered. Editorials, conference posters, letters to the editor, and publications without full-text availability were excluded. During the preparation of this manuscript, the authors used Claude Opus 4.8, Anthropic and Gamma to produce the schematic figures (Figure 1, Figure 2, Figure 3 and Figure 4). The figures are original conceptual illustrations generated with the aid of the tools; they do not depict primary experimental data and do not reproduce, adapt, or incorporate any third-party copyrighted material. All AI-assisted output was subsequently reviewed and verified for scientific accuracy and edited by the authors, who take full responsibility for the content, integrity, and conclusions of the work. No AI tool is listed as an author.

## 3. Trained Immunity and Epigenetic Remodeling of Monocytes in Cirrhosis—How a History of Chronic Injury Forces Innate Immune Cells into Maladaptive States Following Acute Damage

We should start with a conceptual clarification. Trained immunity denotes a heightened, hyperresponsive state of innate immune memory. In contrast, innate immune tolerance, overlapping with the clinical notion of “immune paralysis”, denotes a hyporesponsive state similar to endotoxin tolerance. The two represent opposite poles of innate immune reprogramming. Much of the monocyte phenotype described below in ACLF (reduced HLA-DR expression and blunted TNF-α production on restimulation) is therefore best understood as maladaptive innate tolerance/immune paralysis rather than as classical trained immunity. More broadly, the progression from systemic inflammation to immune paralysis outlined in this and subsequent sections should be read as a synthesizing model: the individual cellular defects are supported by robust human data, whereas their integration into a single, strictly ordered cascade is inferential and remains unestablished as a causal sequence in ACLF.

Available data suggest that the observed mechanisms reflect the immune–tolerogenic nature of the liver, allowing for the control and limitation of excessive inflammatory activation. However, as liver disease progresses, changes occur within the immune system, leading to the development of systemic inflammation and immunodeficiency. Despite persistent immune system activation, cirrhosis-associated immune dysfunction (CAID) causes functional impairments in immune cells and, consequently, an increased risk of infection. Much of the monocyte biology summarised in this section was first characterised in cirrhosis or decompensated cirrhosis and is extrapolated to ACLF unless a specific ACLF cohort is cited [8]. The first groundbreaking report describing monocyte dysfunction in liver cirrhosis was provided by Hassner et al. in 1979 [14]. With the progression of liver disease, the expression of major histocompatibility complex (MHC) class II antigens, specifically human leukocyte antigen D related (HLA-DR), decreases as shown in cirrhosis [15]. One of the proposed causes reported in ACLF cohorts for this reduced HLA-DR expression is an increase in the production of immunosuppressive mononuclear myeloid-derived suppressor cells (CD14+D15- HLA-DR M-MDSCs). Their expansion impairs the capacity of monocytes to present antigens to T lymphocytes; the production of cytokines, including TNF-α and IL-6, in response to bacterial lipopolysaccharide (LPS) declines, and the risk of secondary infections increases. The monocyte ceases to be a cell coordinating immunity, transitioning into a state of active immunosuppression [16]. Studies by Weichselbaum et al. demonstrate that persistent epigenetic changes occur in the monocytes of patients with alcohol-associated hepatitis, impairing the proinflammatory response during stimulation with PAMPs. ACLF, therefore, remains a clinical manifestation of a process that occurred much earlier [17].

Furthermore, there is an increased expansion of monocytes expressing the AXL receptor (CD14+CD16highHLA-DRhigh), which also attenuates the inflammatory response and lymphocyte activation while retaining the capacity for phagocytosis and the clearance of apoptotic cells. Studies indicate that the inhibition of AXL restores the proinflammatory mechanisms of monocytes, which may not only serve as a potential prognostic marker but also act as a target for immunotherapy in patients with liver cirrhosis [18]. Another regulator of monocyte immunosuppression is the increased expression of the MERTK receptor tyrosine kinase on monocytes, which then respond poorly to bacterial lipopolysaccharide and produce fewer proinflammatory cytokines, thereby increasing susceptibility to infections. Simultaneously, MERTK activation on macrophages promotes the progression of fibrosis in chronic liver diseases by inducing a profibrogenic response in hepatic stellate cells [15,19].

These theories are corroborated by a study by Yao et al., in which scRNA sequencing of monocyte subpopulations revealed the most profound genetic changes among proinflammatory monocytes in patients with ACLF compared with a healthy control group. The hemoglobin subunit beta (HBB) and thrombospondin 1 (THBS-1) genes exhibited increased expression. Moreover, THBS-1 expression was higher among fatal ACLF cases, suggesting it may serve as a prognostic factor [20].

## 4. ACLF and Neutrophil Dysfunction—Role of an Impaired Neutrophil Extracellular Trap (NET) Formation, Mitochondrial Distress, and Aberrant C-X-C Motif Chemokine Ligand (CXCL) Chemokine Signaling

In both patients with acutely decompensated cirrhosis (AD) and those with ACLF, the neutrophil count increases while the lymphocyte count decreases. Alterations in the neutrophil transcriptome lead to enhanced granulopoiesis and, consequently, the massive activation of neutrophils, including immature precursors [21]. Despite their hyperactivation, essential functions such as pathogen killing via chemotaxis, phagocytosis, degranulation, neutrophil extracellular traps (NETs), and the generation of reactive oxygen species (ROS) remain impaired [22]. Makkar et al. conducted a detailed evaluation of neutrophil parameters and their association with survival in patients with ACLF. These represent robust, ACLF-specific human data. They demonstrated that as the severity of ACLF increases, the number of neutrophils with a normal mature phenotype (CD16+, CD66b+) decreases, and their phagocytic capacity diminishes, which correlates with increased 90-day mortality. Conversely, the resting oxidative burst of neutrophils is significantly higher in patients with ACLF than in healthy controls and does not increase upon stimulation, which can lead to energy depletion and a weakening of host defense mechanisms, including phagocytosis [23]. In response to the decline in neutrophil phagocytic capacity, there is a compensatory increase in the formation of NETs—webs consisting of released DNA, histones, and granular enzymes such as myeloperoxidase (MPO), which serve as an alternative defense mechanism against pathogens [24]. Upon reaching the failing liver, neutrophils exacerbate the already activated inflammatory process. NETs enhance the release of free radicals and proteolytic enzymes and exacerbate coagulation abnormalities, thereby promoting further hepatocyte apoptosis [25]. As research by von Meijenfeldt et al. indicates, NET formation is active in patients with acute liver failure (a finding established in acute liver failure and other non-hepatic inflammatory models; its direct demonstration in ACLF cohorts is still lacking). Moreover, elevated levels of myeloperoxidase-DNA (MPO-DNA) complexes, a specific marker of NETs, correlate with a poor prognosis [26]. Another study comparing NETosis between AD and ACLF challenges the direct role of extracellular traps in ACLF progression. Blasi et al. demonstrated that the MPO-DNA marker does not differ between patients with AD and ACLF and is not associated with adverse clinical outcomes. In contrast, cfDNA (cell-free DNA originating from the breakdown of various cells, including NETs), IL-6 (an inflammatory marker), and thiobarbituric acid reactive substances [(TBARS), a marker of oxidative stress] remain higher in patients with ACLF than in those with AD and correlate with the severity of organ failure and mortality. This robust, ACLF-specific AD-versus-ACLF comparison tempers the NET-centred account [27].

As shown in an experimental model, another hallmark of neutrophil dysfunction in ACLF is the increased expression of the chemokine ligand CXCL1. By binding to the CXCR2 receptor, it intensifies neutrophil infiltration into the liver, leading to an increase in reactive oxygen species (ROS) production, mitochondrial depolarization, and the activation of caspase-3-dependent apoptotic pathways. Studies indicate that inhibiting CXCL1 may reduce neutrophil recruitment, oxidative stress, and consequently hepatocyte apoptosis, translating into a milder clinical course of ACLF. This pathway has been demonstrated solely preclinically and thus remains inferential for human ACLF [28].

Furthermore, hyperreactive neutrophils in a decompensated cirrhosis and ACLF cohort exhibit an upregulation of the CD177 protein, which enhances their adhesion to endothelial cells, initiating their subsequent massive migration into tissues [29]. They also exhibit excessive reactivity towards parenchymal cells, including hepatocytes, resulting in their damage and death [30].

## 5. ACLF and Metabolic Failure of Immune Cells as a Trigger of Multiorgan Dysfunction—Leukocytes Shift Toward Glycolysis, Lose Oxidative Phosphorylation Capacity, and ROS Bursts That Damage Distant Organs

Profound disturbances in cellular metabolism reflect the adaptive response of immune cells to acute inflammation in ACLF. Peripheral organs, such as the liver, muscles, and adipose tissue, are reprogrammed toward intensified catabolic metabolism (involving proteolysis, glycogenolysis, and lipolysis, among others), which fulfills the heightened energy demands of immune cells [31,32,33]. Concurrently, immune cells shift towards an anabolic metabolism, dictated by the necessity for the intensive synthesis of inflammatory cytokines and chemokines, which promotes enhanced granulopoiesis alongside an overlapping metabolic reprogramming of innate bone marrow cells [21,34]. Metabolic shifts constitute both a consequence of immune cell activation and a primary mechanism determining their differentiation and effector functions [35].

One of the key elements in the pathogenesis of ACLF is the disrupted energy metabolism of leukocytes. This is accompanied by significant alterations in mitochondrial ultrastructure; despite an increase in their numbers, these organelles become smaller, and their cristae become sparser and disorganized [21,36]. Functional derangements also occur, such as a disruption of the tricarboxylic-acid cycle (TCA) at the level of isocitrate dehydrogenase and succinate dehydrogenase. These disruption points are bridged by anaplerotic reactions: glutaminolysis and nucleoside metabolism, which enable continued energy production despite mitochondrial dysfunction [36]. However, energy production via oxidative phosphorylation (OXPHOS) in mitochondria under conditions of an intense inflammatory response remains insufficient. To generate Adenosine Triphosphate (ATP), leukocytes begin to utilize extramitochondrial pathways: aerobic glycolysis and the pentose phosphate pathway (PPP) [21,36,37,38,39]. The PPP not only increases nucleotide production but also leads to an overproduction of nicotinamide adenine dinucleotide phosphate (NADPH), which, serving as a primary donor for the generation of reactive oxygen species, induces oxidative stress and concomitantly exerts a secondary inhibitory effect on mitochondrial OXPHOS [40]. Aerobic glycolysis, known as the Warburg effect, leads to the production of lactate and only 2 ATP molecules per glucose, compared with the 30–32 ATP yielded by OXPHOS. This is energetically inefficient, and the end product additionally exerts various immunomodulatory functions [37,41]. In a state of acute inflammation, lactate exhibits immunosuppressive effects; it promotes the transition of macrophages toward an anti-inflammatory phenotype (a lactate effect characterised largely in sepsis and cancer models and inferred, but not yet directly demonstrated, in ACLF) and inhibits the production of proinflammatory cytokines such as TNF-α and IL-6, as well as T lymphocyte proliferation [39,42,43]. Acting as a signaling molecule rather than simply an end product of metabolism, it plays a crucial suppressive role during inflammation, sepsis, or neoplastic diseases by supporting cellular proliferation [44,45]. Studies confirm that hyperlactatemia is an independent prognostic factor correlating with the degree of liver and other organ failure, and an elevated lactate-to-albumin ratio may serve as a novel biomarker for predicting mortality in ACLF patients [46,47]. Moreover, besides its immunosuppressive action, lactate prolongs inflammation, driving systemic neutrophilia and the mobilization of neutrophils to inflammatory tissues. It activates mobilizers in the bone marrow such as CXCL1, CXCL2, and G-CSF, increases bone marrow vascular permeability via the G protein-coupled receptor 81 (GPR81), and promotes NET formation [48,49]. Simultaneously, it directs macrophages toward transitioning into a fibrotic phenotype, which may further impair systemic organ function [39,50,51]. Therefore, lactate functions as a context-dependent immunometabolite: suppressive on monocytes/macrophages/T cells; pro-inflammatory on bone-marrow myelopoiesis and NETosis via GPR81.

Beyond its role as a free metabolite, lactate also drives histone and protein lactylation—a distinct epigenetic–metabolic modification that reprograms monocyte and macrophage gene expression and mechanistically connects the immunometabolic shift to the trained immunity and epigenetic reprogramming phenomena described earlier [49]. This concept of metabolic control over immune fate has now received direct empirical support from robust, ACLF-specific single-cell data. The 2025 study identified divergent immunometabolic cellular states separating ACLF recovery (ACLF-R) from non-recovery (ACLF-NR), with a stress-induced tolerant state and an oxidative-phosphorylation programme characterizing ACLF-R, in contrast to elevated inflammatory and stress genes [Vimentin (VIM), Lectin, galactoside-binding, soluble, 2 (LGALS2), Triggering Receptor Expressed on Myeloid cells 1 (TREM1)] in ACLF-NR monocytes [52]. Consistent with this, recent integrative reviews now frame ACLF as an exemplar of the metabolic control of immunological processes, highlighting the autotaxin-lysophosphatidic acid axis as a driver of monocyte activation with reduced MERTK and CD163 expression, and synthesizing the emerging multi-omics landscape of the syndrome [53,54]. The immunometabolic rewiring of the ACLF leukocyte is presented in Figure 2. Importantly, the histone lactylation and lactate-signalling data summarised here derive largely from sepsis, cancer and ischaemia models rather than from ACLF, and therefore require direct confirmation in ACLF cohorts.

## 6. The Gut–Liver–Immune Axis in ACLF-Dysbiosis Patterns, Bacterial Translocation, and Pathogen-Associated Molecular Patterns That Shape the Inflammatory Phenotype

A fundamental premise explaining the pathogenesis of ACLF is the theory of systemic inflammation induced by disruptions of the gut–liver–immune axis, which encompasses complex interactions between the gastrointestinal tract and hepatic sinusoids, mutually connected via the portal circulation and the biliary system [55]. Changes occurring in the cirrhotic liver, such as portal hypertension, impaired bile flow, and secondary intestinal mucosal ischemia, along with the loss of tight junctions in enterocytes, predispose to the compromise of the intestinal barrier and subsequent bacterial translocation through portal and lymphatic routes [56,57,58]. Furthermore, as liver disease progresses, significant quantitative and qualitative alterations occur in the gut microbiota. Dysbiosis involves not only bacterial overgrowth but, above all, a disruption of species diversity, featuring the loss of commensal microbes and the expansion of potentially pathogenic species [59]. Mantovani et al., in a study of a cirrhosis cohort, demonstrated that fecal bacterial diversity decreases alongside the progression of liver disease, which simultaneously correlates with MELD (Model for End-Stage Liver Disease) and Child–Pugh scores, as well as the risk of 90-day mortality [60]. Regardless of the etiology, cirrhosis is associated with an increase in *Fusobacteria*, *Proteobacteria*, *Enterococcaceae*, and *Streptococcaceae* with a relative decrease in *Bacteroidetes*, *Ruminococcus*, *Roseburia*, *Veillonellaceae* and *Lachnospiraceae* [61]. Changes within bacterial taxa in ACLF are presented in Table 2.

Molecules of bacterial origin, referred to as pathogen-associated molecular patterns (PAMPs), including lipopolysaccharide (LPS), peptidoglycans, lipopeptides, and fragments of bacterial DNA, play a substantial role in the pathogenesis of ACLF [55,63]. Upon reaching the liver via the portal and systemic circulation, they bind to Toll-like receptors (TLR4 and TLR9) on Kupffer cells and hepatic stellate cells (HSCs), inducing a proinflammatory phenotype and increasing the production of IL-6 and TNF-α [57,64]. One of the pioneering studies on gut microbiota and ACLF demonstrated a decline in the *Ruminococcaceae* and *Lachnospiraceae* families, which was associated with an increase in proinflammatory cytokines [TNF-α, IL-6, Interleukin 2 (IL-2)]. It should be noted, however, that this is an association rather than a demonstrated causal effect; it is at most consistent with a protective role for these taxa. Any biomarker or probiotic application therefore remains a hypothesis that would require prospective, ACLF-specific validation before translation [65]. Further evidence supporting the theory of systemic inflammation was provided by Zang et al.’s study on HBV-ACLF, which revealed an observed increase in bacteria from the *Burkholderiaceae* family and a positive correlation between them and the chemokine IP-10, which acts as a chemoattractant recruiting immune cells (primarily Th1 T lymphocytes, Natural Killer (NK) cells, and monocytes) to inflammatory foci [68].

Alongside the intensification of inflammation, defensive factors become depleted. The reduction in bacteria from the *Lachnospiraceae* and *Ruminococcaceae* families, coupled with the simultaneous overgrowth of potentially invasive taxa, including *Enterobacteriaceae* and *Streptococcaceae*, is associated with a decrease in short-chain fatty acids (SCFAs) such as acetate, propionate, and butyrate. These compounds control immune cell differentiation, provide nutritional support, and exert beneficial effects on the intestinal barrier [57,71,72,73]. The excessive proliferation of opportunistic pathogens such as *Enterococcus* and *Klebsiella* also induces structural damage to enterocyte microvilli [66].

A profound dysregulation of bile acid (BA) metabolism occurs during the course of ACLF. Under physiological conditions, the gut microbiota converts primary BAs into secondary ones (e.g., ursodeoxycholic acid [UDCA] and lithocholic acid [LCA]), which effectively attenuate the systemic inflammatory response through the activation of Takeda G-protein-coupled receptor 5 (TGR5) and Farnesoid X receptor (FXR). The capacity to synthesize these protective molecules is strictly dependent on commensal taxa, such as *Clostridium* and *Ruminococcus*, which undergo depletion in ACLF. The bile–acid conversion pathway is established in gut-microbiota biology and extrapolated to ACLF. The taxa depletion is reported in an HBV-ACLF cohort [69,70]. Furthermore, research by Bao et al. demonstrated a significant deficit of *Parabacteroides distasonis* in ACLF patients compared with patients with AD. In preclinical studies in animal models, *Parabacteroides distasonis* has been shown to increase the pool of secondary BAs and restore FXR pathway stimulation; these findings provide only preclinical proof of concept and have not yet been evaluated in patients with ACLF [70].

Two cautions apply to this microbiome evidence. First, the reported taxonomic associations are largely at the association level and are cohort-, geography-, and etiology-dependent. They do not by themselves establish causality. Second, the microbiome restoration strategies discussed here, including those targeting Parabacteroides distasonis, are preclinical (animal models) and should be regarded as hypothesis-generating rather than as established therapies. However, disturbances regarding dysbiosis in ACLF remain insufficiently understood, and most studies to date encompass gut–liver axis disorders in chronic liver diseases or liver cirrhosis. Nevertheless, initial studies assessing the prognostic value of the microbiota in the course of ACLF are emerging. Solé et al. demonstrated that species such as *Paraprevotella clara*, *Bacteroides salyersiae*, *Clostridium* sp., and *Roseburia hominis* are associated with a favorable prognosis, whereas *E. faecium*, *Streptococcus thermophilus*, and *Ruminococcus lactaris* are linked to a higher risk of death [67]. The observed increase in bacteria from the *Pasteurellaceae* family also correlates with increased mortality [65]. Combining microbiome analysis with established clinical scores such as MELD is a plausible way to improve prognostic accuracy, but this remains a proposal that has not been prospectively validated in ACLF cohorts [65,67]. Research by Yao et al. in a cohort of HBV-ACLF patients also illustrates how dysbiosis and a reduction in bacterial diversity translate into changes in serum biomarkers such as ALT, AST, total bilirubin, and INR. A reduced abundance of the *Bacteroidetes* phylum was shown to correlate with an increase in alpha- fetoprotein (AFP), while an increase in *Veillonella* was associated with elevated total bilirubin values. These correlations support a significant role of the microbiota in the development and course of ACLF [66]. Although gut dysbiosis is commonly observed in patients with liver cirrhosis, the specific overgrowth of bacteria from the *Proteobacteria* phylum is a strong predictor of the onset of ACLF and secondary multiorgan failure, particularly renal injury, in patients with chronic liver disease [62]. Serum metabolomic analysis indicates that an increase in bacteria-derived metabolites (including aromatic compounds, secondary or sulfated bile acids, and benzoate) is a strong risk marker for ACLF development and short-term mortality [74]. Despite the varying aspects evaluated in the discussed studies, they consistently agree on the drastic decline in microbiota profile diversity. The extent to which this loss is a passive marker rather than an active contributor to disease progression remains uncertain, given the largely associative nature of the available data, which requires causal confirmation in ACLF.

## 7. Systemic Endothelial Dysfunction in ACLF: A Silent Instigator of Kidney Injury, Circulatory Collapse, and Cerebral Edema

As a consequence of the mechanisms described above, damage and dysfunction of the vascular endothelium occur in patients with ACLF. Studies in decompensated cirrhosis and ACLF confirm the presence of CD177 gene and protein overexpression in neutrophils, which induces their potent adhesion to endothelial cells [29]. This results in endothelial damage, the activation of local prothrombotic processes, and the release of plasma markers of vascular dysfunction, which may secondarily serve as a critical pathophysiological mechanism in the development of multiorgan failure over the course of ACLF [29,75]. Another indicator of endothelial dysfunction during inflammation is the elevation of endocan levels—a biomarker that modulates leukocyte migration by binding to their Lymphocyte Function-Associated Antigen-1 (LFA-1) receptors and inhibiting interactions with vascular Intercellular Adhesion Molecule 1 (ICAM-1) [76]. The level of this protein is elevated as early as the stage of hepatic steatosis and fibrosis, whereas in advanced cirrhosis, it closely correlates with the risk of developing organ failure. Because this evidence derives primarily from steatosis, fibrosis, and general cirrhosis cohorts rather than from dedicated ACLF studies, it provides a strong mechanistic basis to hypothesize, rather than to draw final conclusions regarding a significant role for endothelial dysfunction in the pathogenesis of ACLF [77,78]. A phenomenon equally closely linked to the progressive impairment of the vascular barrier in ACLF is the pathological increase in the angiopoietin-2 to angiopoietin-1 ratio. Angiopoietins are proteins that play an important role in angiogenesis. Angiopoietin-1 (Ang-1) exerts a vasoprotective effect, supporting cell differentiation and suppressing inflammation, whereas angiopoietin-2 (Ang-2) competitively blocks TIE receptors for Ang-1, exacerbating pathological angiogenesis and promoting inflammatory processes [79]. The clinical significance of this phenomenon is corroborated by prospective cohort studies providing compelling evidence, albeit derived from patients with decompensated cirrhosis. Although the defined cohort did not strictly comprise patients with ACLF, a proportion may have met the diagnostic criteria, though this was not assessed in the study. Allegretti et al. demonstrated that high Ang-2 levels strongly correlate with increased mortality and, as an independent marker, exhibit predictive value for 90-day mortality equal to or higher than classical prognostic scores (MELD, CLIF-C ACLF-CLIF Consortium Acute-on-Chronic Liver Failure score). Furthermore, elevated Ang-2 precipitates a more severe course of acute kidney injury (AKI), perfectly illustrating how endothelial dysfunction impacts the development of multiorgan failure—a mechanism of direct relevance to ACLF, though here demonstrated outside a strictly ACLF-defined cohort [80]. The ligand imbalance (increase in Ang-2 relative to Ang-1) stimulates the production of hepatocyte growth factor (HGF), which in turn enforces the prolonged activation of the CCAAT/enhancer-binding protein beta (C/EBPβ) transcription factor in the liver. Although this cascade mimics early organ regeneration at the molecular level, under the conditions of ACLF, the sustained overexpression of C/EBPβ permanently suppresses metabolic genes and disrupts hepatocyte differentiation, directly driving the progression of acute liver failure. This mechanism was defined in an experimental model in which sepsis was used as the precipitant to induce ACLF. It is therefore directly pertinent to ACLF pathophysiology but remains inferential until confirmed in human ACLF [81].

Findings from other studies demonstrate that the impairment of the regenerative angiocrine functions of liver endothelial cells stems from the reduced expression of HGF regulated by the C-X-C motif chemokine receptor 7 (CXCR7), C-X-C motif chemokine receptor 4 (CXCR4), and Inhibitor of DNA binding 1 (ID1) genes. In the course of ACLF, there is a progressive deterioration of the CXCR7-ID1-HGF-dependent pathway. Although under physiological conditions, mesenchymal stromal cells stimulate the endothelium to express CXCR7, ID1 and HGF, this mechanism is critically disrupted in the pathological environment of ACLF. This leads to a substantial depletion of the CXCR7+ endothelial cell subpopulation, subsequently resulting in a decline in the total pool of endothelial cells and a drastic inhibition of hepatocyte proliferation. Unlike the other endothelial mechanisms discussed in this section, this pathway was characterised directly in ACLF rather than extrapolated from cirrhosis or experimental models; it therefore represents the endothelial finding here most firmly anchored in ACLF-specific data [82].

Studies conducted on a rat model of microsurgical hepatic cholestasis aimed at analyzing the cerebral vasculopathy accompanying hepatic encephalopathy (HE) have shown that vascular disturbances in the course of ACLF result from the increased expression and activity of key endothelial enzymes. The excessive activation of endothelial nitric oxide synthase (eNOS), inducible nitric oxide synthase (iNOS), and cyclooxygenase-2 (COX-2) leads to the non-physiological release of vasodilatory factors: nitric oxide (NO) and prostacyclin (PGI2), with a concurrent lack of significant changes in the concentration of the vasoconstrictive thromboxane A2 (TXA2). The resulting distorted secretory profile of the endothelium induces pathological vasodilation and an increase in cerebral blood flow, initiating a cascade of neurological damage, including cerebral edema and hypoxia, which may ultimately lead to HE or secondary ischemia of the central nervous system. Because this evidence derives from an animal model, its extrapolation to ACLF should be regarded as a mechanistic hypothesis requiring prospective validation in dedicated ACLF cohorts rather than as established clinical evidence [83].

In summary, endothelial dysfunction in ACLF has a multifactorial etiology. Bacterial translocation (PAMPs) from a compromised intestinal barrier and the release of molecules from disintegrating cells (DAMPs) induce a generalized activation of innate immunity, triggering subsequent pathophysiological mechanisms. At the hepatic level, secondary inflammation and oxidative stress enforce the pathological capillarization of liver sinusoidal endothelial cells (LSECs) and stimulate inflowing neutrophils to form NETs, exacerbating intrahepatic microthrombosis [84]. Conversely, at the circulatory level, a massive secretion of vasodilatory mediators [e.g., NO, Carbon Monoxide (CO), PGI2] occurs. This pathological secretory profile results in extreme vasodilation of the splanchnic and systemic vascular beds, causing arterial hypotension and a critical drop in effective circulating blood volume. In response to central hypovolemia, a compensatory, intensified activation of vasoconstrictive systems ensues. This phenomenon drastically worsens tissue ischemia and serves as the direct driving force behind secondary multiorgan failure, including hepatorenal syndrome acute kidney injury (HRS-AKI) and encephalopathy [85].

## 8. The Liver–Spleen Axis in ACLF

The circuit outlined above does not terminate in the liver. ACLF is precipitated by insults such as bacterial infection or alcohol-associated hepatitis that provoke an excessive systemic inflammatory response, and the spleen is anatomically interposed in this response: its venous outflow drains directly into the portal system, while portal hypertension renders it congested, enlarged and immunologically remodeled [86].

Splenic immune activation is an underappreciated contributor. Gut-derived microbial products that escape hepatic clearance through portosystemic shunts reach the splenic compartment, where resident and recruited macrophages add to the circulating pool of TNF-α and IL-6, so that the spleen behaves as a second, extrahepatic amplifier of the cytokine storm rather than a passive reservoir [86]. The fibrogenic arm of this axis has now been demonstrated directly: in fibrotic liver injury, a discrete subset of CD11b+CD43^hi^Ly6C^lo^ splenic monocytes is mobilized, migrates into the liver, converts into monocyte-derived macrophages and activates hepatic stellate cells, whereas splenectomy attenuates fibrogenesis [87]. A complementary mechanism operates through spleen-derived LIGHT/TNFSF14, which acts on hepatic macrophages via the lymphotoxin-β receptor (LTβR) and c-Jun N-terminal kinase (JNK) signaling to increase TGF-β1 production and thereby drive stellate-cell activation in the same profibrogenic macrophage–stellate cell circuit described above for MERTK^+^ macrophages [19,88]. In this sense, the spleen supplies the liver with fibrogenic myeloid cells and with the signals that instruct them.

Splenomegaly and hypersplenism close the loop at the haemodynamic level. Increased splenic venous return sustains portal hypertension and the hyperdynamic, vasodilated circulation of decompensated cirrhosis, while hypersplenic sequestration produces thrombocytopenia and leukopenia, aggravating the coagulation failure and the susceptibility to infection that characterize organ failure in ACLF [8,85,86].

ACLF-specific evidence, however, remains scarce and so far radiological: in 278 patients with HBV-ACLF, the CT-derived liver-to-spleen volume ratio (LSVR) showed that both too low and too high values are associated with 28- and 90-day mortality [89]. Consistent with the framing adopted throughout this review, the spleen is therefore best regarded as a plausible pathogenic amplifier in ACLF—fueling systemic inflammation, sustaining portal hypertension and feeding fibrogenic monocytes to the liver whose causal role still awaits prospective validation in dedicated ACLF cohorts.

## 9. Therapeutic Implications: Liver Transplantation and Immune-Modulating Approaches, Who Do They Help?

ACLF, characterized by multiorgan failure and high short-term mortality, currently lacks a specific targeted therapy. Treatment relies on the elimination of triggering factors, the prevention of complications, and ultimately liver transplantation, which significantly improves prognosis. At the same time, the identification of the main pathophysiological mechanisms of this disorder, including the generalized inflammatory response and immunological dysfunction, presents an opportunity to integrate novel therapeutic strategies. The disease trajectory and therapeutic windows in ACLF are presented in Figure 3.

The therapeutic evidence discussed here can be grouped into three tiers:

(a) Established: such as liver transplantation, which remains the only intervention with a clear survival benefit, alongside organ-specific supportive care;
(b) Promising but investigational: such as therapeutic plasma exchange, G-CSF, mesenchymal stem cells, and anti-cytokine agents, which remain adjunctive and rest on conflicting or early-phase data;
(c) Mechanistic: hypotheses requiring prospective validation and including the immunometabolic and phase-targeted strategies emphasized above.

### 9.1. Liver Transplantation and the Transplant Window

In contrast to the immunomodulatory strategies discussed below, liver transplantation remains the only intervention with a robust survival signal in ACLF and constitutes the central thrust of the 2023 EASL clinical practice guidance, whose core management framework comprises early triage, control of the precipitating event, multiorgan support, timely definition of futility, and structured transplant evaluation [4,90]. On this basis, transplantation should be actively considered in severe presentations—including HBV-related ACLF with a MELD score above 30, or persistent ACLF grade 2–3 despite adequate antiviral therapy and organ support—provided that futility criteria are not met. The 2025 APASL Kyoto Consensus formalizes a finite ‘transplant window’ situated between the reversible ‘Golden Therapeutic Window’ and the point of therapeutic futility, underscoring that the survival benefit of transplantation is critically time-dependent and that delayed referral represents a principal, modifiable cause of avoidable mortality [2]. A pathophysiology-driven review would therefore be clinically incomplete without positioning transplantation, rather than any single immunomodulator, as the current benchmark against which adjunctive therapies must be judged.

### 9.2. Immune-Modulating Strategies

One of the proposed therapeutic options has been the administration of G-CSF, a pleiotropic factor that stimulates the migration of bone marrow stem cells (CD34+) to the damaged liver parenchyma, which facilitates their differentiation into hepatocytes and promotes direct organ regeneration [91]. Initial studies from Asian centers demonstrated potential for G-CSF in patients with viral HBV-related ACLF [92,93]. After 3 months of follow-up, the survival rate in the G-CSF group was 48.1%, compared with a mere 21.4% in the control group [92]. It was also demonstrated that in the case of decompensated liver cirrhosis without features of ACLF, G-CSF therapy improves prognosis [94,95]. However, the largest European multicenter randomized controlled trial (GRAFT) revealed a complete lack of clinical benefit. G-CSF administration improved neither overall survival nor transplant-free survival, and an increased risk of adverse events was additionally observed in patients receiving the factor [96]. A comprehensive meta-analysis of randomized controlled trials proves that in patients with decompensated liver cirrhosis, the use of G-CSF contributes to a reduction in mortality and a decreased risk of sepsis, particularly in the Asian population; however, such evidence is lacking in the case of ACLF [97]. This discrepancy in results between Asian and European patients may stem from the differing etiologies of ACLF depending on location and the varying diagnostic criteria applied across different regions [98].

Furthermore, the therapeutic potential of G-CSF in the course of ACLF is strictly dependent on the pathophysiological phase of the disease. In patients in the fully developed, hyperinflammatory stage or with active infection, this intervention may exacerbate the further recruitment of neutrophils and monocytes, promoting the “cytokine storm.” Conversely, in the advanced phase, associated with immune paralysis and the inhibition of liver cell regeneration, it emerges as a potential therapeutic opportunity. Further research is necessary to determine specific immunological mechanisms across defined stages of liver disease [99].

Human serum albumin (HSA) also demonstrates significant therapeutic potential; its classic oncotic, antioxidant, and scavenging properties are utilized in patients with liver cirrhosis to prevent hemodynamic instability induced by paracentesis, as well as to prevent the development of ascites and hepatorenal syndrome [100]. Casulleras et al. described novel immunomodulatory and anti-inflammatory properties of albumin. Undergoing active internalization into the endosomal compartment within leukocytes, it directly inhibits signaling pathways dependent on TLRs. This intracellular mechanism effectively reduces the secretion of proinflammatory cytokines without compromising the proper phagocytic and antimicrobial functions of the immune system [11]. Moreover, albumin supplementation in patients with decompensated cirrhosis restores the normal morphology and function of endothelial cell mitochondria [101]. Studies confirm that therapeutic plasma exchange utilizing a 5% albumin solution (PE-A5%) in patients with ACLF goes beyond the simple replenishment of the vascular bed, effectively restoring deficient antioxidant, binding, and detoxification capabilities. Furthermore, this intervention optimizes the patient’s condition, improving hemodynamic parameters and the functioning of the liver, kidneys, and central nervous system, as well as reducing the severity of the systemic inflammatory response. The beneficial effect is evidenced by the reduction in the MELD score, which may ultimately translate into a significant increase in survival within this patient cohort [102].

Despite its confirmed safety profile and documented immunomodulatory properties, other studies indicate that intravenous albumin supplementation in patients with ACLF does not yield a statistically significant improvement in terms of survival, reduction in new infections, or the prevention of renal dysfunction. The limited efficacy of standard infusion in suppressing the advanced inflammatory cascade suggests that extracorporeal techniques, such as plasmapheresis, might prove to be a much more effective strategy for harnessing the therapeutic potential of this protein [103,104]. This inference should, however, be drawn cautiously, because the dedicated plasma-exchange evidence stream is more nuanced than a simple superiority claim. The APACHE phase III programme evaluates plasma exchange with 5% albumin (PE-A5%) in ACLF grades 1b/2/3a against a 90-day survival endpoint, while a 2026 single-centre randomized controlled trial reported a markedly lower 28-day mortality with therapeutic plasma exchange than with standard care (44.3% versus 63.9%) but no significant difference in 90-day mortality. The consistent pattern across meta-analyses is that plasma exchange improves survival in acute liver failure, whereas in randomized ACLF subgroups it confers, at most, a short-term rather than a durable survival advantage; the statement that extracorporeal techniques are “much more effective” should therefore be regarded as provisional pending the completion of adequately powered ACLF trials [105,106].

Evidence for albumin as a specific treatment for ACLF remains limited: many trials are small, non-randomized, or extrapolated from decompensated liver cirrhosis rather than well-defined ACLF cohorts. Its role as a disease-modifying therapy for ACLF itself continues to be investigated [100,107].

Therapy based on mesenchymal stem cells (MSCs) exhibits considerable potential in the treatment of liver diseases due to their pleiotropic immunomodulatory and antifibrotic properties, as well as their ability to stimulate endogenous hepatocyte regeneration [108]. Randomized clinical trials involving patients with HBV-related ACLF have confirmed a statistically significant improvement in survival rates, alongside a more rapid normalization of bilirubin levels and a decline in MELD scores in patients treated with intravenous infusions of bone marrow-derived MSCs [109]. It should be noted, however, that available meta-analyses provide inconsistent conclusions regarding the assessment of specific endpoints. The team of Wang et al. demonstrated that patients with ACLF derive markedly greater benefits from cellular therapy than patients with decompensated liver cirrhosis. A favorable impact on the MELD score, a decrease in total bilirubin concentration, and an increase in albumin levels were also observed, though without a discernible effect on aminotransferase activity or coagulation parameters [110]. Conversely, another study demonstrated, in addition to a similar effect on basic organ function parameters, an improvement in coagulation indices and a reduction in the risk of complications such as hepatic encephalopathy and gastrointestinal bleeding. Importantly, these positive biochemical and clinical changes did not ultimately translate into prolonged overall survival in the analyzed patient group [111]. Recent reports confirm the general beneficial impact of MSCs on patients’ biochemical profiles; however, researchers strongly emphasize the urgent need to standardize medical protocols. Precisely determining the optimal cell dose, the most effective route of administration, and identifying the appropriate timing to initiate therapy remain significant challenges [112]. Despite these limitations, MSC infusions are considered a safe form of intervention even in severe forms of ACLF, although patients in the early, milder stages of the disease appear to benefit the most from them [113]. Immunomodulatory therapies in ACLF are summarized in Table 3.

The phase-targeted therapeutic map for ACLF is presented in Figure 4. It should be emphasized that this phase-targeted framework is a mechanistic, hypothesis-generating construct intended to arrange current understanding. It is not a clinically validated or guideline-endorsed treatment algorithm, and prospective trials are required before it can be introduced to medical practice. 

## 10. Future Perspectives

Several priorities emerge from this review. Definitions and trial endpoints should be standardized across consortia based on the harmonized 2025 APASL Kyoto and EF-CLIF frameworks. Mechanisms currently inferred from cirrhosis or non-hepatic inflammatory models, such as immunometabolic reprogramming, histone lactylation, NET formation, and the microbiome–bile–acid axis, require further validation in dedicated ACLF cohorts, ideally with a focus on distinct phenotypes such as HBV-ACLF. Biomarker-guided stratification of the dominant immune state could enable phase-targeted trial designs. Adequately powered randomized controlled trials are needed for plasma exchange, G-CSF, and mesenchymal stem cell therapy before any can be recommended as standard adjuncts to liver transplantation.

## 11. Conclusions

ACLF remains one of the most difficult challenges in modern hepatology, characterized by an acute clinical course and dramatically high short-term mortality. As demonstrated in this paper, the pathogenesis of this syndrome constitutes a complex network of interdependent processes extending far beyond the mere injury of the hepatic parenchyma. The summarised sequence should be read as a synthesizing model assembled from partially overlapping mechanistic hypotheses and heterogeneous evidence rather than a fully validated linear pathway. The pivotal driver of multiorgan failure in the course of ACLF is a devastating inflammatory cascade initiated by gut–liver axis disruptions, bacterial translocation, and massive cell breakdown. The spleen, congested and immunologically remodeled by portal hypertension, emerges from this analysis as a plausible accomplice in that cascade rather than a passive victim of it, although this remains to be confirmed in ACLF-specific cohorts. This state rapidly evolves towards profound immune paralysis, culminating in generalized endotheliopathy, which is thought to contribute to microcirculatory collapse and secondary organ failure in a ‘silent’ yet catastrophic manner. Despite progress in understanding the molecular and cellular foundations of ACLF, the development of effective treatments remains a clinical challenge. It is useful to distinguish established evidence from what is still investigational or hypothetical. As established by clinical evidence, the convergent 2025 APASL Kyoto and EF-CLIF definitions and prognostic scores now allow ACLF to be recognised and risk-stratified at the bedside, while treatment of the precipitant, organ support and, in selected patients, liver transplantation remain the mainstays of management. A second tier comprises the still-investigational, cell- and cytokine-directed strategies examined in this review, such as granulocyte colony-stimulating factor, mesenchymal stromal cells and anti-cytokine agents. The discussed immunomodulatory strategies, while promising in preclinical studies and early clinical phases, yield inconclusive results in the broader population. This is most likely due to patient heterogeneity, etiological disparities, and the failure to synchronize interventions with the appropriate pathophysiological phase of the disease. Only the rigorous standardization of definitions and research protocols, along with the personalization of therapy based on the patient’s current immunological profile, will make it possible to overcome past therapeutic failures and to translate current pathophysiological knowledge into clinical practice. A third tier consists of the mechanistic hypotheses developed throughout this review—maladaptive innate immune reprogramming (tolerance/immune paralysis rather than classical trained immunity), immunometabolic reprogramming, lactate signalling and histone lactylation, and the gut-microbiome–bile–acid axis—that still require prospective validation in dedicated ACLF cohorts. This call for precision medicine is no longer purely aspirational: the 2025 single-cell demonstration of distinct recovery (ACLF-R) and non-recovery (ACLF-NR) immunometabolic states provides an empirical, cell-resolved substrate for stratifying patients and timing immunomodulatory or extracorporeal interventions to the dominant immune state [52]. Anchoring future trials to the harmonized 2025 APASL Kyoto and EF-CLIF frameworks and to such immunometabolic signatures offers the most credible route from mechanistic understanding to effective, phase-matched therapy [2,7,52].

## Figures and Tables

**Table 1:** Definitional frameworks for ACLF, reframed against the APASL Kyoto typology.

Consortium	Underlying Population	Organ-Failure Basis	Role of Infection	Grading/Score	28-Day Mortality Range	Kyoto Type (A/B)
APASL/AARC	Chronic liver disease with or without cirrhosis; large non-cirrhotic fraction; HBV reactivation and alcohol dominate; no prior decompensation required	Liver failure obligatory (bilirubin ≥ 5 mg/dL + INR ≥ 1.5) with ascites and/or HE within 4 weeks; extrahepatic failures appear later	Infection treated as a consquence/complication—not an accepted defining precipitant (sepsis-driven cases largely excluded)	AARC score (bilirubin, HE, INR, lactate, creatinine) → grades I–III	Grade-dependent: I ≈ <20%, II ≈ 40–50%, III ≈ >75% (approx., cohort-dependent)	Predominantly Type A (single hepatic insult on non-decompensated CLD; potentially reversible)
EASL-CLIF (CANONIC/EF-CLIF; CLIF-C ACLF)	Acute decompensation of cirrhosis (prior or index decompensation required)	CLIF-C Organ Failure score across 6 systems (liver, kidney, brain, coagulation, circulation, respiration); organ failure defines ACLF	Bacterial infection is a major precipitant in Western cohorts; recognised as both precipitant and consequence	CLIF-C ACLF score; grades 1–3 by number of organ failures	ACLF-1 ≈ 22–23%, ACLF-2 ≈ 30–32%, ACLF-3 ≈ 70–77% (28-day)	Mixed—frequently Type B (deterioration on decompensated cirrhosis); Type A when a clear precipitant is present
NACSELD	Hospitalised patients with cirrhosis (framework derived from infected cirrhotic cohorts)	≥2 extrahepatic organ failures of 4 (shock, grade III/IV HE, renal replacement, mechanical ventilation); hepatic failure not required	Infection central—a key precipitant; framework built around infected cirrhotics	Binary definition (≥2 extrahepatic OF = ACLF); no graded score	≥2 OF ≈ 30–50%; rises steeply per added OF (up to >90% with 4)	Typically Type B (infection-precipitated deterioration on decompensated cirrhosis)
COSSH (HBV-ACLF)	HBV-related chronic liver disease, cirrhotic and non-cirrhotic; HBV reactivation/flare	Modified CLIF-OF adapted to HBV; admits hepatic failure on non-cirrhotic CLD; COSSH-ACLF II score	HBV reactivation is the dominant precipitant; infection a frequent consequence	COSSH-ACLF/COSSH-ACLF II score; grades 1–3	Grade-dependent, steep gradient (≈1: ~20% → 3: >70–80%)	Predominantly Type A (HBV-flare insult); bridges APASL and EASL concepts

**Abbreviations:** AARC, APASL ACLF Research Consortium; ACLF, acute-on-chronic liver failure; AKI, acute kidney injury; APASL, Asian Pacific Association for the Study of the Liver; CANONIC, CLIF Acute-oN-chrONic lIver failure in Cirrhosis study; CLIF-C, Chronic Liver Failure Consortium; CLD, chronic liver disease; COSSH, Chinese Group on the Study of Severe Hepatitis B; EASL, European Association for the Study of the Liver; EF-CLIF, European Foundation for the study of Chronic Liver Failure; HBV, hepatitis B virus; HE, hepatic encephalopathy; INR, international normalised ratio; NACSELD, North American Consortium for the Study of End-Stage Liver Disease; OF, organ failure. Kyoto type: Type A = ACLF on non-decompensated (compensated) chronic liver disease, precipitant-driven and potentially reversible; Type B = ACLF superimposed on already decompensated cirrhosis. Mortality figures are approximate and cohort-dependent. Sources: CANONIC/EASL-CLIF [1,3,4]; APASL update [5] and the APASL Kyoto consensus (2025 [2]); COSSH [6]; NACSELD [7].

**Table 2:** Gut-microbiome shifts in ACLF and their prognostic direction.

Taxon	Direction	Functional Consequence	Prognostic Association	Reference
*Proteobacteria * (phylum)	↑	↑ PAMP/endotoxin load; strong driver of barrier failure	Adverse—strong predictor of ACLF onset and secondary renal failure	[57,62,63,64]
*Enterobacteriaceae*	↑	↑ LPS load, endotoxaemia; ↓ barrier integrity	Adverse—worse outcome; linked to ACLF development and AKI	[58,62,65]
*Streptococcaceae*	↑	Pathobiont overgrowth; pro-inflammatory milieu	Adverse—associated with severity	[61,65]
*Enterococcus*/*Enterococcaceae* (incl. *E. faecium*)	↑	Enterocyte microvillus damage; translocation	Adverse—*E. faecium* associated with higher mortality	[66,67]
*Veillonella*	↑	Bile–acid/metabolic shift	Adverse—correlates with elevated total bilirubin	[66]
*Burkholderiaceae* (HBV-ACLF)	↑	Positive correlation with IP-10 (immune-cell chemoattractant)	Adverse—pro-inflammatory recruitment	[68]
*Pasteurellaceae*	↑	Dysbiotic expansion	Adverse—correlates with increased mortality	[65]
*Lachnospiraceae*	↓	↓ SCFA (butyrate); impaired barrier and immune regulation	Protective loss—reduction tracks ↑ TNF-α/IL-6 and poorer outcome	[61,65]
*Ruminococcaceae*	↓	↓ SCFA and secondary bile–acid synthesis (↓ FXR/TGR5)	Protective loss—adverse	[65,69]
*Clostridium/* *Ruminococcus* (commensal BA converters)	↓	↓ secondary bile acids (UDCA, LCA) → ↓ TGR5/FXR anti-inflammatory signalling	Protective loss—adverse	[69,70]
*Parabacteroides* *distasonis*	↓	↓ secondary BA pool/FXR stimulation (deficit vs. AD)	Adverse; therapeutic restoration increases secondary BAs (promising)	[70]
*Bacteroidetes * (phylum)	↓	Reduced diversity	Adverse—reduction correlates with rising AFP	[66]
*Paraprevotella clara*, *Bacteroides salyersiae*, *Clostridium* sp., *Roseburia hominis*	present	SCFA producers; barrier support	Favorable—presence associated with better prognosis	[67]
Overall faecal α-diversity	↓	Global loss of protective metabolic function; correlates with MELD/Child–Pugh	Adverse—lower diversity predicts ↑ 90-day mortality	[60,65]

**Abbreviations:** AD, acute decompensation; AFP, alpha-fetoprotein; AKI, acute kidney injury; BA, bile acid; FXR, farnesoid X receptor; IP-10, interferon-γ-induced protein 10; LCA, lithocholic acid; LPS, lipopolysaccharide; MELD, Model for End-stage Liver Disease; PAMP, pathogen-associated molecular pattern; SCFA, short-chain fatty acid; TGR5, Takeda G-protein-coupled receptor 5; UDCA, ursodeoxycholic acid; ↑, increase; ↓, decrease. Direction is relative to healthy controls/acute-decompensation comparators. “Protective loss” denotes depletion of a beneficial taxon, which is prognostically adverse.

**Table 3:** Immunomodulatory therapies in ACLF: evidence ledger.

Therapy	Proposed Mechanism	Best Evidence (*n*, Design)	Outcome	Asian vs. Western Divergence	Recommendation
G-CSF	CD34+ progenitor mobilisation → hepatic regeneration; myeloid functional modulation	Duan 2013 (*n* ≈ 55, RCT) [92] GRAFT 2021 (*n* = 176, RCT) [96] Di Martino 2023, (meta-analysis) [97]	Asian trials: ↑ survival (3-month 48% vs. 21%, Duan). GRAFT: no overall or transplant-free survival benefit, ↑ adverse events	Marked benefit largely confined to Asian (HBV) cohorts; absent in European (alcohol-predominant) ACLF; etiology and criteria differ	Not recommended for routine ACLF use (EASL 2023); phase-dependent rationale; investigational
Human serum albumin (intravenous)	Oncotic expansion; antioxidant/scavenging; endosomal TLR-signal inhibition; endothelial mitochondrial restoration	ATTIRE 2021 (*n* = 777, RCT) [104]	No reduction in infection, renal dysfunction or death as targeted IV therapy; benefit confined to classicalindications; safe	Limited divergence; principal RCT evidence Western; ACLF-specific confirmatory data lacking globally	Recommended for SBP, HRS-AKI and large-volume paracentesis; NOT a standalone disease-modifying ACLF therapy
Plasma exchange/PE-A5%(extracorporeal)	Removal of circulating PAMPs/DAMPs, cytokines and bilirubin; restoration of functional albumin and detoxification	Fernández 2024 (proof-of-concept study) [102] Swaroop 2026 (*n* = 194, RCT) [106]	Signal for improved 28-day survival/organ function (TPE 44.3% vs. 63.9% mortality); no consistent 90-day ACLF benefit; strongest established benefit in ALF	Active investigation in both regions; APASL incorporates PE in selected HBV-ACLF algorithms; Western RCT data emerging	Promising/emerging; not yet standard of care; reasonable transplant bridge in selected centres; under RCT evaluation
Mesenchymal stromal cells (MSC)	Immunomodulatory, anti-fibrotic and pro-regenerative paracrine signalling	Lin 2017 (*n* ≈ 110, RCT) [109] Wang 2023 (meta-analysis) [110] Liu 2022 (meta-analysis) [111] Lu 2025 (meta-analysis) [112]	Consistent biochemical improvement and survival benefit in HBV-ACLF RCTs (Lin); meta-analyses inconsistent on hard endpoints; greatest benefit early-stage; safe	Most RCT evidence Asian (HBV-ACLF) (Lin); dose/route/timing heterogeneity limits generalisability	Investigational; not standard; protocol standardisation required
Anti-cytokine/immunometabolic (emerging)	IL-6/TNF-α blockade; lactate–lactylation modulation; PD-1/TIM-3 checkpoint inhibition to reverse immunoparalysis; AXL/MERTK targeting	Preclinical and early-phase only; no completed phase III in ACLF Naveau 2004 (*n* = 36, RCT) [114] Boetticher 2008 (*n* = 48, RCT) [115] Tan 2025 [116] Bao 2025 [117] Markwick 2015 (*n* = 48, prospective study) [118] Bernsmeier 2015 (*n* = 119, prospective study) [119]	No clinical efficacy data; theoretical phase specific use (anti-cytokine in hyper-inflammation; checkpoint blockade in immunoparalysis) Anti–TNF-α agents (infliximab, etanercept) showed harm in severe AH underscoring the risk of unselected cytokine blockade	Not applicable (preclinical)	Experimental; not for clinical use outside trials

**Abbreviations**: AH, alcohol-associated hepatitis; ALF, acute liver failure; ATTIRE, Albumin To prevenT Infection in chronic liveR failurE; BM-MSC, bone-marrow-derived mesenchymal stromal cell; G-CSF, granulocyte colony-stimulating factor; GRAFT, G-CSF to treat ACLF trial; HBV, hepatitis B virus; HRS-AKI, hepatorenal syndrome–acute kidney injury; IL, interleukin; PAMP/DAMP, pathogen-/damage-associated molecular pattern; PE, plasma exchange; PE-A5%, plasma exchange with 5% albumin replacement; RCT, randomised controlled trial; SBP, spontaneous bacterial peritonitis; TLR, Toll-like receptor; TNF-α, tumour necrosis factor α; TPE, therapeutic plasma exchange; ↑, increase; →, leads to. Liver transplantation remains the only definitive therapy and the survival benchmark; the therapies above are adjunctive or investigational. Recommendation status reflects EASL 2023 guidance [4] and the APASL Kyoto consensus (2025) [2].

## Data Availability

No new data were created or analyzed in this study. Data sharing is not applicable to this article.

## Abbreviations

AARC	APASL ACLF Research Consortium
AASLD	American Association for the Study of Liver Diseases
ACLF	Acute-on-chronic liver failure
ACLF-R	Acute-on-chronic liver failure recovery
ACLF-NR	Acute-on-chronic liver failure non recovery
AD	Acute decompensation
AFP	Alpha-fetoprotein
AH	Alcoholic hepatitis
AKI	Acute kidney injury
Ang-1	Angiopoietin-1
Ang-2	Angiopoietin-2
APASL	Asian Pacific Association for the Study of the Liver
ATP	Adenosine Triphosphate
BA	Bile acid
CAID	Cirrhosis-associated immune dysfunction
CANONIC	CLIF Acute-oN-chrONic lIver failure in Cirrhosis study
cfDNA	cell-free DNA
CLD	Chronic liver disease
CLIF-C	Chronic Liver Failure Consortium
COSSH	Chinese Group on the Study of Severe Hepatitis B
CO	Carbon Monoxide
COX-2	Cyclooxygenase-2
CXCL1	C-X-C motif chemokine ligand 1
CXCL2	C-X-C motif chemokine ligand 2
CXCR4	C-X-C motif chemokine receptor 4
CXCR7	C-X-C motif chemokine receptor 7
C/EBPβ	CCAAT/enhancer-binding protein beta
DAMPs	Damage-associated molecular patterns
EASL	European Association for the Study of the Liver
EASL-CLIF	European Association for the Study of the Liver—Chronic Liver Failure Consortium
EF-CLIF	European Foundation for the study of Chronic Liver Failure
eNOS	Endothelial nitric oxide synthase
FXR	Farnesoid X receptor
G-CSF	Granulocyte colony-stimulating factor
GPR81	G protein-coupled receptor 81
HBB	Hemoglobin subunit beta
HBV	Hepatitis B virus
HE	Hepatic encephalopathy
HGF	Hepatocyte growth factor
HLA-DR	Human leukocyte antigen D Related
HRS-AKI	Hepatorenal syndrome-acute kidney injury
HSA	Human serum albumin
HSCs	Hepatic stellate cells
ICAM-1	Intercellular Adhesion Molecule 1
ID1	Inhibitor of DNA binding 1
IFN-γ	Interferon gamma
IL-1β	Interleukin 1 beta
IL-2	Interleukin 2
IL-6	Interleukin 6
IL-8	Interleukin 8
IL-10	Interleukin 10
iNOS	Inducible nitric oxide synthase
INR	International normalised ratio
IP-10	Interferon-γ-induced protein 10
JNK	c-Jun N-terminal kinase
LCA	Lithocholic acid
LFA1	Lymphocyte Function-Associated Antigen-1
LGALS2	Lectin, galactoside-binding, soluble, 2
LPS	Lipopolysaccharide
LSECs	Liver sinusoidal endothelial cells
LSVR	Liver-to-spleen volume ratio
LTβR	Lymphotoxin-β receptor
MELD	Model for End-stage Liver Disease
MERTK	MER receptor tyrosine kinase
MHC	Major histocompatibility complex
M-MDSCs	Mononuclear myeloid-derived suppressor cells
MPO	Myeloperoxidase
MPO-DNA	Myeloperoxidase- DNA
MSCs	Mesenchymal stem cells
NACSELD	North American Consortium for the Study of End-Stage Liver Disease
NADPH	Nicotinamide adenine dinucleotide phosphate
NETs	Neutrophil extracellular traps
NF-κB	Nuclear factor kappa B
NK	Natural Killer
NLRs	NOD-like receptors
NO	Nitric oxide
OF	Organ failure
OXPHOS	Oxidative phosphorylation
PAMPs	Pathogen-associated molecular patterns
PD-1	Programmed cell death protein 1
PE	Plasma exchange
PE-A5%	Plasma exchange with 5% albumin replacement
PGI2	Prostacyclin
PPP	Pentose phosphate pathway
PRRs	Pattern recognition receptors
RCT	Randomised controlled trial
ROS	Reactive oxygen species
SBP	Spontaneous bacterial peritonitis
SCFA	Short-chain fatty acid
SI	Systemic inflammation
TBARS	Thiobarbituric Acid Reactive Substances
TREM1	Triggering Receptor Expressed on Myeloid cells 1
TCA	Tricarboxylic-acid cycle
TGR5	Takeda G-protein-coupled receptor 5
THBS-1	Thrombospondin 1
TIM-3	T-cell immunoglobulin and mucin-domain containing-3
TLRs	Toll-like receptors
TNF-α	Tumor necrosis factor alpha
TPE	Therapeutic plasma exchange
TXA2	Thromboxane A2
UDCA	Ursodeoxycholic acid
VIM	Vimentin
